# Iodine as Essential Nutrient during the First 1000 Days of Life

**DOI:** 10.3390/nu10030290

**Published:** 2018-03-01

**Authors:** Inés Velasco, Sarah C. Bath, Margaret P. Rayman

**Affiliations:** 1 Pediatrics, Obstetrics and Gynecology Unit, Hospital de Riotinto, Avda La Esquila 5, 21660 Minas de Riotinto, Huelva, Spain; 2 Department of Nutritional Sciences, Faculty of Health and Medical Sciences, University of Surrey, Guildford GU2 7XH, UK; s.bath@surrey.ac.uk (S.C.B.); m.rayman@surrey.ac.uk (M.P.R.)

**Keywords:** iodine, deficiency, neurodevelopment, behavioural disorders, foetal programming

## Abstract

Iodine is an essential micronutrient incorporated into thyroid hormones. Although iodine deficiency can lead to a broad spectrum of disorders throughout life, it is most critical in the early stages of development, as the foetal brain is extremely dependent on iodine supply. During the last two decades, our understanding of thyroid physiology during gestation has substantially improved. Furthermore, thyroid hormone receptors have been identified and characterised in placental and embryonic tissues, allowing us to elucidate the maternal-foetal transfer of thyroid hormones. Experimental studies have demonstrated that the cyto-architecture of the cerebral cortex can be irreversibly disturbed in iodine deficiency causing abnormal neuron migratory patterns which are associated with cognitive impairment in children. In this context, the role of iodine as key factor in the programming of foetal and infant neurodevelopment, needs to be revisited with a special focus on areas of mild to moderate iodine deficiency. The objective of this review is to summarize the available evidence from both animals and human studies, for the effect of iodine deficiency (particularly, of maternal hypothyroxinemia) on brain development and neurological or behavioural disorders, such as lower intelligence quotient (IQ) or attention deficit hyperactivity disorder (ADHD).

## 1. Introduction

Thyroid hormones intervene directly or indirectly in many metabolic and developmental processes such as thermal and metabolic regulation, somatic growth and development and function of the central nervous system (CNS) [1,2]. Iodine requirements during gestation increase to fulfil both foetal needs and altered maternal thyroid physiology [3]. 

Iodine is considered to be an essential micronutrient as it is obtained exclusively through diet or iodine supplements, and it cannot be replaced by any other nutrient in human development [4]. This essentiality becomes even more obvious in the early stages of intrauterine life since adequate iodine intake in pregnancy is needed to achieve optimal foetal neurodevelopment [2,5]. The foetus, followed by the young child, is the most vulnerable to iodine deficiency (ID) [6].

For years, it was believed that ID was a problem restricted to certain geographic areas and specific individuals (e.g., malnourished) and that it resulted in a well-defined clinical spectrum (hypothyroidism, goitre and brain damage) [7]. However, the re-emergence of iodine deficiency in some industrialized countries has reawakened concern about the cognitive consequences of this deficiency [8,9]. 

Foetal programming is a concept that links nutritional and environmental conditions during embryonic and foetal development with risk of diseases in later life [10]. The first 1000 days of life have been established as a “window of opportunity” for potential interventions able to determine crucial pathways of human growth and development [11].

The aim of this review is to summarize the current knowledge from both animals and human studies on iodine deficiency as a key factor in foetal programming, particularly, on brain development and neurological or behavioural disorders, such as lower intelligence quotient (IQ) or attention deficit hyperactivity disorder (ADHD).

## 2. Methods

We searched MEDLINE, EMBASE and Web of Science using the following MeSH terms as inclusion criteria: iodine, iodine supplementation, children, foetus, neurodevelopment, brain, cognitive function. These results were then divided into experimental studies (summarised in Table 1) and studies in humans. 

An additional search was performed to assess the potential effectiveness of iodine supplementation during pregnancy and childhood on cognitive and neuropsychological outcomes, including only the information obtained from systematic reviews and meta-analysis (Table 2). Since the inclusion criteria was the study design, the review included information from areas of mild to moderate iodine deficiency as well as severe iodine deficiency. Eleven studies met the inclusion criteria and there were no studies to exclude. 

### 2.1. Thyroid Physiology in Pregnancy

Pregnancy is accompanied by significant changes in thyroid function, resulting from a complex combination of factors specific to gestation that together stimulate the maternal thyroid gland [12]. During the first half of gestation, human chorionic gonadotropin produced by the placenta has an effect resembling that of thyroid-stimulating hormone (TSH) (due to the structural homology between the molecules) and acts to stimulate directly the maternal thyroid [1,12]. During this period, the foetal thyroid is inactive so the foetus is entirely dependent on thyroxine of maternal origin [13,14]. Despite the fact that the foetal thyroid begins to function from 18 to 20 weeks of gestation [15], iodine supply still remains solely through the mother.

Pregnancy involves a higher demand for thyroid hormones [1,12]. In healthy pregnant women with an adequate iodine intake, the thyroid gland regulates the release of hormones to achieve a new balance and maintains this balance until the end of the gestational process [16]. In general, the higher hormone need can only be met by a proportional increase in hormone release, which depends directly on the intake of iodine through the diet [5,8].

The adaptation to increased iodine nutritional needs is achieved without difficulty by the thyroid gland when intrathyroidal iodine stores are sufficiently replete [3]. Conversely, when the thyroid gland responds inadequately (due, for instance, to iodine deficiency), such changes in thyroid requirements may not be adequately met and adaptation mechanisms cannot succeed [16]. Obviously, the more severe the iodine deficiency, the more serious will be the foetal and maternal consequences [6,7]. An inadequate thyroidal response has even been shown to occur in healthy pregnant women residing in areas where iodine deficiency is not greater than mild-to-moderate [17,18].

### 2.2. Changing the Paradigm of Pre-Natal Iodine Deficiency

Since the first epidemiological studies conducted by Pharoah [19] and Thilly [20] in the 1970s, the association between severe iodine deficiency in pregnant women and foetal neurological damage has been extensively reviewed and demonstrated in the scientific literature. 

For a long time, it was believed that the main factor responsible for alterations in foetal neurological development was maternal hypothyroidism (defined as elevated serum TSH concentrations with low free thyroxine (FT4) in the early stages of pregnancy [42]. Thus, when a pregnant woman was found to have normal thyroid function, neurodevelopmental alterations in the foetus were thought to be unlikely.

**Figure 1:**
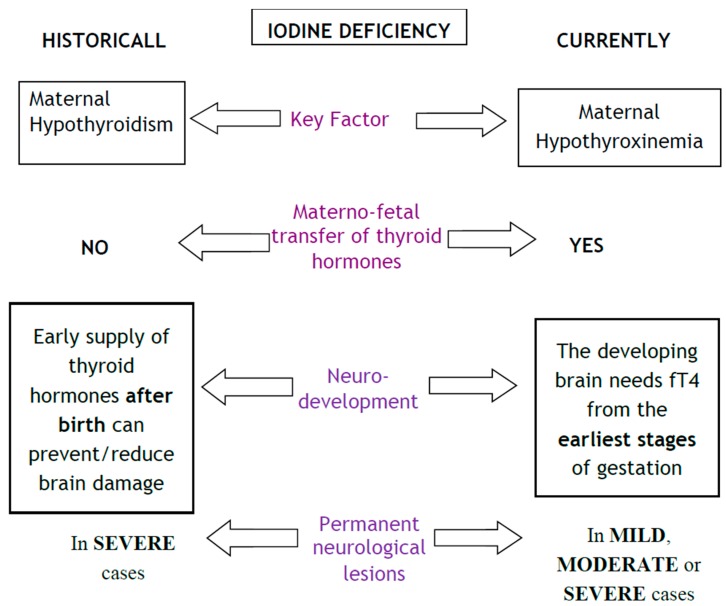
Foetal and neonatal effects of iodine deficiency during pregnancy. The main advance has been that maternal hormone transfer to the foetus during pregnancy is definitely accepted, as well as the existence of damage in the progeny even in the absence of maternal hypothyroidism.

However, for the last two decades, epidemiological and experimental studies have demonstrated that foetal neurodevelopment is not only affected when the mother is hypothyroid, but also when she is “hypothyroxinemic” in the early stages of pregnancy [21,43,44]. Isolated hypothyroxinemia in pregnancy is defined as the presence of a free thyroxine (FT4) value below the 2.5th percentile with a thyrotropin (TSH) level within the reference range [45]. The damage is caused by decreased availability of maternal T4 to the developing brain. Figure 1 shows the differences between the classic and current understanding of the physiology of prenatal iodine deficiency. 

### 2.3. Maternal–Foetal Transference of Iodine and Thyroid Hormones

In all mammalian species, the placenta actively transports iodide from the maternal to the foetal circulation to provide iodide for thyroid hormone synthesis [2]. 

Under normal conditions, embryonic tissues have a set of security mechanisms to protect their development. Some of these mechanisms are physical barriers (placenta and ovular membranes) that avoid free transfer of maternal thyroid hormones to the foetus, preventing it from being exposed to the same plasma fluctuations that occur in the maternal bloodstream [46,47]. Another security mechanism is the presence of deiodinase enzymes in the placenta and foetal cerebral tissues [48]. Deiodinase enzymes, particularly type 2 (DIO2) present in the developing brain, convert maternal fT4 to tri-iodothyronine (T3), since the direct transfer of maternal T3 is extremely low [46,48]. 

In nutritional iodine deficiency, the organism activates self-regulating mechanisms such that T3 is synthesized preferentially over T4 as a way to conserve iodine [49,50]. This leads to maternal hypothyroxinemia, where plasma T4 levels fall, but circulating T3 and TSH levels remain normal [45,51].

Maternal hypothyroxinemia appears in healthy pregnant women (without any clinical signs or underlying thyroid pathology) and indicates maternal inability to transfer adequate T4 to the embryo for its proper neurological development [45].

Maternal hypothyroxinemia during the first half of gestation has been associated with permanent and irreversible neurological alterations in the embryo and the foetus [44]. Experimental animal studies have contributed to a better characterization of cerebral areas affected by inadequate availability of fT4 (Table 1) [21,22,23,24,25,26,27,28,29,30,31].

Traditionally, it was thought that maternal hypothyroxinemia was exclusively due to a dietary intake of iodine that was inadequate to meet iodine needs during pregnancy. However, recent studies have demonstrated the existence of maternal hypothyroxinemia even in iodine-sufficient areas [52,53,54], possibly in relation to environmental endocrine disrupters, drugs or auto-immune thyroid disease [44]. Whatever the cause, the insufficient supply of T4 to developing neural tissues seems to be the basis of adverse permanent cognitive and or behavioural sequels in the progeny.

### 2.4. Foetal Neurological Development and Consequences of Prenatal Iodine Deficiency

In humans, cerebral cortical development occurs between the 6th and the 24th week of gestation [55]. Thyroid hormones are involved either directly or indirectly in most of the neurodevelopmental processes of the embryo and the foetus [2,56]. This explains why thyroid deficiency in early pregnancy causes irreversible effects. 

Thyroid hormone receptors have been shown to be expressed profusely both in neurons and in glial cells (astrocytes and oligodendrocytes) [57]. At the neuronal level, T3 binds to a thyroid- hormone receptor, activating gene transcription; this favours the expression of certain patterns of genes involved in axonal and dendrite outgrowth, and in synapse formation, myelination, cell migration, and proliferation of specific cell populations [57,58]. For proper neuronal organization (e.g., synaptic transmission, laminar cytoarchitecture of the cerebral cortex), appropriate interaction with glial cells is required. It is clear that foetal neurodevelopment follows a very precise and constrained sequence of events [57]. The response period of the cell is called “competence” [59]. The same cell will not respond before or after this period. It is also apparent that the maturation sequence is not formed by a succession of independent events, but rather by a cascade where each anomalous event will affect subsequent development.

**Figure 2:**
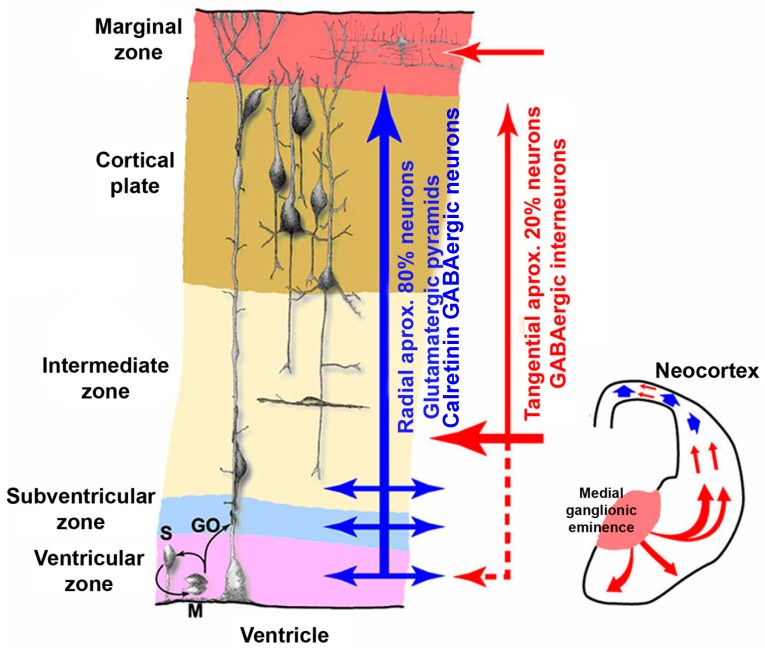
The neuronal migration process is affected by moderately low thyroid hormone levels in early foetal life. The figure shows a disorganized cortical plate where both the radial (blue arrow) and tangential (red arrow) migratory pathways are distorted by maternal hypothyroxinemia (courtesy of Berbel P).

It is clear then, that any situation compromising maternal thyroid hormone transfer to the foetus will disturb the neuronal migration process. As a result, neurons will not reach their final destination in the upper layers and their abnormal positioning will cause alterations in the laminar architecture of the cerebral cortex [60]. In biopsies performed in experimental animals, maternal thyroid hormone deficiency was found to cause permanent and irreversible lesions in the cerebral-cortex cytoarchitecture [21]. As with gestational hypothyroidism, hypothyroxinemia causes blurred neocortical layering (Figure 2).

Once the almost universal involvement of thyroid hormones in the development and proliferation of foetal neural tissue is recognised, it is not difficult to foresee the complex spectrum of neurologic disorders that can be associated with iodine deficiency at the early stages of intrauterine development. The permanent lesions of the cerebral cortex, hippocampus and cerebellum will offer relatively well-defined characteristics:

- lack of damage at the encephalic trunk or spinal cord will prevent direct motor symptoms, but motor coordination will be altered [60];
- lesions will affect higher-order integrative cortical areas with a poorly defined anatomical basis, including silent areas of the associative cortex [61];
- there will be no clinical expression during the perinatal period, with later onset of symptoms during infancy or school age [57];
- such lesions can hardly be detected by the current techniques for prenatal diagnosis such as ultrasounds or foetal MRI [62].

### 2.5. The Evolving Picture of Brain Damage Due to Iodine Deficiency

Important population changes (such as salt-iodization programmes, strategies of iodine supplementation or fortification and even silent iodine prophylaxis) have contributed to a progressive eradication of the most severe clinical features of perinatal iodine deficiency [5,6]. The epidemiology of ID has evolved from goitre and mental disability to a new clinical spectrum of neuropsychological disorders associated with maternal hypothyroxinemia [63,64].

Different reviews and meta-analyses have attempted to quantify the effect of iodine deficiency on cognitive and neuropsychological development in children (Table 2) [8,32,33,34,35,36,37,38,39,40,41]; however, the conclusions differ significantly between reviews as a result of: (i) the inclusion of studies from regions with both severe and mild-to-moderate ID; (ii) differences in study inclusion criteria, with some reviews focusing on the effect of iodine deficiency whereas others evaluated the impact of iodine supplementation or fortification; (iii) different tests used to measure developmental domains; and (iv) simply the year that the review was carried out. It is clear that changes in iodine nutritional status (e.g., from the application of universal salt iodization) over recent decades have substantially modified the significance of the effect of iodine deficiency or indeed of supplementation in targeted populations.

The most recent study, a randomised, double-blind, placebo-controlled trial of iodine supplementation in mildly iodine-deficient pregnant women from Thailand and India found no effect on child neurodevelopment at age 5–6 years [65]. It should be pointed out, however, that although the median urinary iodine concentration (131 µg/L) classified the women as iodine deficient, in one of the countries (India), pregnant women were actually iodine sufficient (median 188 µg/L); furthermore, both Thailand and India are countries with iodized salt programmes where the general population is iodine-sufficient. It is therefore likely that the women will have entered pregnancy with sufficient thyroidal iodine stores to supply their own needs and those of their foetuses over the course of the pregnancy [65,66].

Considering iodine deficiency as urinary iodine concentration (UIC) below 150 µg/L in pregnant women, two observational studies performed in the UK and Australia have evaluated associations with offspring cognition [43,67]. In the ALSPAC cohort, children of iodine-deficient mothers (defined as iodine-to-creatinine ratio below 150 µg/g) had a significantly higher risk of suboptimal cognitive outcomes on the subscales of verbal IQ, reading accuracy and reading comprehension at 8–9 years, as well as a mean total IQ that was lower by 3.4 points [67]. In Australia, children born to mothers with UIC below 150 µg/L had lower spelling scores at the age of 9 years, though the association with grammar and English literacy were attenuated after adjusting for maternal occupation and education [43]. These findings persisted in adolescence in spite of the children growing up in an iodine-replete environment following the introduction of mandatory fortification of bread with iodized salt in 2009 [68]. By contrast, the Generation R cohort in The Netherlands, which is iodine-replete, did not find a significant relationship between maternal low UIC and children’s non-verbal IQ or language comprehension [69], although the effect sizes were similar to those of the ALSPAC study. 

More recently, a population-based observational study from Norway (MoBA) showed that low maternal iodine intake (below the Estimated Average Requirement of 160 µg/day) during pregnancy was associated with child language delay, behaviour problems and reduced fine motor skills at the age of three years [44]. It was notable that iodine supplementation during pregnancy did not show a protective effect [44].

In spite of amelioration of the iodine status in the general population of many countries, we are witnessing a new scenario where impaired cognitive outcomes are augmented by a myriad of behavioural disorders such as attention-deficit/hyperactivity disorder (ADHD) or autism [54,70]. The evidence currently available indicates an increased risk of ADHD in the offspring of mothers with abnormal serum thyroid hormone concentrations during early pregnancy; ADHD has been described in cases of hyperthyroidism [71] iodine deficiency [72] maternal hypothyroxinemia [73] and mild thyroid-hormone insufficiency [74]. The MoBA study in Norway found that a low iodine intake (<200 µg/L) was associated with higher ADHD symptom scores, but not with the diagnosis of ADHD [75]. Although iodine supplements during pregnancy were more effective than levothyroxine (LT4) in a pilot study aimed at improving neuro-intellectual outcomes [76], they did not reduce the risk of ADHD [75]. Indeed, the MoBA study showed a negative association between multivitamin/mineral supplements containing iodine and ADHD-like symptoms [75].

Additionally, severe maternal hypothyroxinemia in early gestation has been consistently associated with offspring autistic symptoms [77]. Other subtle psychopathological symptoms have been described in areas of marginal ID, i.e., deficits in inhibition, working memory and global executive functioning in children of mothers with low UIC [78].

This spectrum of neuropsychological disorders can be understood through a developmental biology approach [51], where the structural alterations of cortical lamination observed in experimental animal models may help to explain the development of behavioural and mental disorder throughout the life course [79]. 

### 2.6. Iodine Deficiency and Foetal Programming

Iodine deficiency during the early stages of human development leads to antenatal consequences that share similarities with neural tube defects (NTD) due to folate deficiency:

- for both entities, the nutritional predisposing conditions are known;
- the mechanisms that trigger morphological alterations are known; in both cases, the neuronal migration process is disturbed. In the case of NTD, neurones are stopped in their migration to the neural crest, whereas in iodine deficiency, neurones are stopped in their migration to the upper layers of cerebral cortex (Figure 2).
- an effective prophylaxis is available, ideally from preconception to the end of neurogenesis.

From the very early stages of pregnancy, iodine requirements and the fT4 levels in maternal serum are modified; the existence of maternal hypothyroxinemia in the first trimester interferes directly with embryogenesis and foetal neurodevelopment. Additionally, recent evidence suggests that maternal hypothyroxinemia has an epigenetic effect in the offspring that potentiates the regulated expression of certain genes through mRNA transcription and/or the downregulation of specific micro RNAs (miRNAs) [80]. These facts suggest that ID acts as a chronic nutritional deficiency aggravated by pregnancy and maternal hypothyroxinemia that can imprint cells of central nervous system of the offspring and exert effects postnatally.

### 2.7. Iodine Deficiency during Early Childhood

In addition to pregnant and lactating women, children under two years of age have been identified as vulnerable groups for ID by WHO-UNICEF-International Council for the Control of Iodine Deficiency Disorders (ICCIDD) [66].

New evidence supports a life-course perspective on childhood development with adverse early experiences having long-term physiological and epigenetic effects on brain development and cognition [81]. In this regard, iodine plays a pivotal role that substantially contributes to postnatal development and plasticity of neural tissues [82]. 

Although the effects of prenatal ID cannot be completely overcome by iodine supplementation in children [43,68,83], intervention trials have shown that providing iodine alone or in combination with other micronutrients in populations with concurrent deficiencies [84] can provide infants and young children with adequate micronutrient requirements.

A recent randomised, double-blind, placebo-controlled trial compared the effectiveness of direct iodine supplementation in infants with indirect iodine supplementation (by providing iodine supplements to breastfeeding mothers) and found that the latter was more effective [85]. In areas of moderate-to-severe ID without effective iodised salt programmes, iodine supplementation for lactating mothers should be therefore considered [85].

“Nurturing care” is the term that has been coined to include nutritional, environmental and emotional support to promote the development of key brain regions that will have lifelong benefits, including improved health and wellbeing, and increased ability to learn and earn [86]. It seems obvious that adequate iodine intake is essential for this goal to be reached, but it should be realised that the effectiveness of iodine supplementation is dependent on its use during multiple and overlapping critical time-windows when development of specific capacities and abilities can most powerfully be enhanced [87].

## 3. Summary

Iodine is an essential nutrient, particularly crucial for the neurodevelopment. In spite of the limitation of a non-systematic review, the main strength of this review is that we have gathered the most substantial information from both animals and human studies, in order to provide a fuller understanding of the role of iodine in brain development and the potential consequences of its deficiency at the early stages of human life.

Pregnancy involves a higher demand for thyroid hormones [1,12], which may not be adequately met even in healthy pregnant women residing in areas where iodine deficiency is not greater than mild-to-moderate [17,18]. Foetal neurodevelopment is not only affected when the mother is hypothyroid, but also when she is “hypothyroxinemic” in the early stages of pregnancy and studies have demonstrated the existence of maternal hypothyroxinemia even in iodine sufficient areas. In all mammalian species, the placenta actively transports iodide from the maternal to the foetal circulation to provide iodide for thyroid hormone synthesis [2]. Any situation compromising maternal thyroid hormone transfer to the foetus might lead to permanent lesions of the cerebral cortex, hippocampus and cerebellum. In spite of amelioration of the iodine status in the general population of many countries, we are witnessing a new scenario where impaired cognitive outcomes are augmented by a myriad of behavioural disorders such as attention-deficit/hyperactivity disorder (ADHD) or autism [54,70]. In summary, Iodine deficiency acts as a chronic nutritional deficiency aggravated by pregnancy and maternal hypothyroxinemia that can imprint cells of central nervous system of the offspring and exert effects postnatally. Iodine also plays a pivotal role that substantially contributes to postnatal development and plasticity of neural tissues [82].

## Figures and Tables

**Table 1:** Experimental studies demonstrating brain areas affected by maternal hypothyroxinemia.

	Study Design	Structural Alterations	Functional or Clinical Consequences
Lavado-Autric (2003) [21]	Rat dams fed a low iodine diet	Significant proportion of neurons found at locations that were aberrant or inappropriate with respect to birth date	Alteration in foetal brain histogenesis and cytoarchitecture might explain cognitive impairment in the progeny
Ausó (2004) [22]	Inducement of mild and transient hypothyroxinemia in rat dams by methimazole (MMI)	The cytoarchitecture and the radial distribution of neurons was significantly affected in the somatosensory cortex and hippocampus	Increased frequency of abnormal responses to acoustic stimulus Susceptibility to audiogenic seizures
Opazo (2008) [23]	Inducement of maternal hypothyroxinemia in rat dams by MMI	A significant reduction in the capacity of the brain for spatial learning Impaired dendrite and synapse stability Detrimental changes in long-term potentiation, affecting cognitive processes	Impaired learning capacity, prolonged latency of learning process
Babu (2011) [24]	Rat dams were fed a low iodine diet and given 1% KClO4 in drinking water (to lower the iodine content in the thyroid gland)	Significant decrease in myelin basic protein (MBP) and mitochondrial gene for cytochrome c oxidase III (Cox III) levels during neocortical development Increased number of apoptotic neurons distributed in all the layers of the neocortex	Thyroid hormone responsiveness in postnatal cortex is more sensitive to decrease in T4 than T3 concentration
Pinazo-Durán (2011) [25]	A rat model of controlled thyroid hormone deficiency	Delayed glial development and myelination in optic nerve	Reduction in the volume of the eye and optic nerve cross-sectional area Thinning of the retinal layers
Wei (2013) [26]	Four groups of rat dams: control group, mild ID, severe ID and MMI-treatment group	Impaired growth of axonal-related proteins Delayed axonal growth in hippocampus Damage of the morphological axon in the developing hippocampus	The deficits in axonal development might promote axonal regeneration in the hippocampus, but this process might not fully compensate for the damage induced by low thyroxine.
Gilbert (2014) [27]	Rat dams were exposed to propylthiouracil (PTU) in their drinking water to inhibit the thyroid hormone synthesis	Presence of subcortical-band heterotopia (SBH), a type of neuronal migration error resulting in neurones, oligodendrocytes and microglia in the corpus callosum of the offspring.	SBH in humans is an important type of malformation often associated with intractable epilepsy of childhood.
Wang (2014) [28]	A maternal hypothyroxinemia model (using mild ID diet) and two maternal hypothyroidism models (through a severe ID diet and MMI water respectively)	Reduced proliferation of cerebellar granule neuron precursors (CGNPs) Decreased total dendritic length of Purkinje cells (the most important neurons in the cerebellum)	Affected motor coordination and motor activity in which the cerebellum plays a critical role.
Cisternas (2016) [29]	Inducement of maternal hypothyroxinemia in rat dams by MMI	Affected synaptic protein distribution and impaired neuronal function. This deleterious effect is dependent on astrocyte and neuron integrity.	Affected neuronal plasticity which is dependent on interplay between astrocytes and neurons.
Gilbert (2016) [30]	Rat dams were exposed to propylthiouracil (PTU) in their drinking water to inhibit thyroid hormone synthesis	Reduced expression of neurotrophins that are important for neural processing. Restricted activity-dependent induction of neuroplasticity in the hippocampus. Changes persisted into adulthood despite the return to euthyroidism.	Altered structural and functional pathways in both the developing and adult brain.
Opazo (2017) [31]	Inducement of maternal hypothyroxinemia in rat dams by MMI	Unbalanced reactivity of microglia (decreased) and astrocytes (increased) to inflammatory stimuli.	Astrocytes could react strongly in inflammation, inducing neuronal death in the central nervous system.

**Table 2:** Reviews and meta-analyses of the effect of iodine deficiency (ID) on cognitive and neuropsychological development.

	Year	*N* of Studies	*N* of Subjects	Comments	Conclusions
Bleichrodt [32]	1994	21 18	2676 2214	Systematic review (21 studies) and meta-analysis (18 studies). Observational and intervention studies carried out from 1969 to 1991 were pooled	A number of studies point to a negative effect of ID on cognitive development in children and adults from seriously ID areas, but other studies do not clearly show such an effect. Meta-analysis: The difference between iodine-deficient and non-ID groups is 13.5 IQ points.
Verhoef [33]	2003	12 15	-- --	Meta-analysis Observational and intervention studies were analysed separately.	Observational studies indicate that ID is associated with impaired cognitive development. ID in the first half of pregnancy is irreversible.
Qian [34]	2004	37	12,291	Meta-analysis of Chinese studies Analysis of observational studies, intervention studies both during and after pregnancy.	The damage to the intelligence of children exposed to severe ID was profound, demonstrated by a 12.5 IQ point loss; children recovered 8.7 IQ points with iodine supplementation or iodine sufficiency during and after pregnancy.
Melse-Boonstra [35]	2010	7	615	Review of controlled trials (most of them randomized) of iodine supplementation in children.	Iodine supplementation in school-aged children can reverse certain delays in cognitive performance. Iodine supplementation in early life may be more beneficial than supplementation at school age.
Skeaff [36]	2011	8	844	Review of intervention studies carried out in pregnant women in areas of mild-to-moderate ID.	There is a need for well-designed trials to determine the effect of iodine supplementation in mildly to moderately iodine-deficient pregnant women on child neurodevelopment.
Trumpff [8]	2013	7 5 5	3660 425 935	Three different reviews (all of European studies) of the effect on children’s cognitive/psychomotor development of: - maternal hypothyroxinemia. - neonatal hyperthyrotropinaemia. - odine supplementation.	It is difficult to establish a direct link between maternal ID and maternal hypothyroxinemia, as well as between maternal ID and elevated neonatal TSH levels at birth. Some studies suggest that iodine supplementation from the first trimester until the end of pregnancy may decrease the risk of cognitive and psychomotor developmental delay in the offspring.
Bougma [37]	2013	2 8 9 4	147 1943 2027 2441	Systematic review and meta-analysis. Four different analyses: RCTs with iodine supplementation of mothers (2 studies) Non-RCTs with iodine supplementation of mothers and/or infants (8 studies) Prospective cohort study stratified by pregnant women’s iodine status (9 studies) Prospective cohort study stratified by newborn iodine status (4 studies)	Iodine deficiency has a substantial impact of mental development. Average effect sizes were 6.9 to 10.2 IQ points lower in ID children than in iodine replete children. Quantifying more precisely the contribution of ID to delayed mental development in young children requires more well-designed RCTs, including trials on the role of iodized salt.
Zhou [38]	2013	2 6	19,683 719	Systematic review. 2 RCTs conducted in severe ID areas and 6 RCTs in mild-to-moderate ID regions.	Iodine supplementation during pregnancy or the peri-conceptional period in regions of severe ID reduced the risk of cretinism, but there were no improvements in childhood intelligence, gross development, growth or pregnancy outcomes, although there was an improvement in some motor functions.
Taylor [39]	2014	17	641	Systematic review and meta-analysis. 9 RCTs and 8 observational studies of iodine supplementation during pregnancy from mild-to-moderate ID regions.	Iodine supplementation improves some maternal thyroid indices and may benefit aspects of cognitive function in school-age children, even in marginally ID areas.
Lam [40]	2017	2	494	Systematic review. RCTs that evaluate the effect of iodine on cognitive performance or academic performance among children aged 4–18 were included.	Iodine supplementation achieved a significant improvement in non-verbal fluid intelligence in ID children but no significant change in memory.
Taylor [41]	2017	3	507	Systematic review and Meta-analysis. RCTs of iodine intervention during pregnancy.	There was no significant difference between the intervention and control groups for child cognition in any of the RCTs.
